# Delayed Presentation and Treatment of Sagittal Synostosis: A Case Report

**DOI:** 10.7759/cureus.47178

**Published:** 2023-10-17

**Authors:** Amy V Lyons, Kelechi C Eseonu, Stefan Kluzek

**Affiliations:** 1 Medical Sciences Division, University of Oxford, Oxford, GBR; 2 Orthopaedics, Guys and Saint Thomas' NHS Foundation Trust, London, GBR; 3 Sports and Exercise Medicine, Nuffield Department of Orthopaedics, Rheumatology and Musculoskeletal Sciences, University of Oxford, Oxford, GBR; 4 Sports and Exercise Medicine, Queen’s Medical Centre, University of Nottingham, Nottingham, GBR

**Keywords:** delayed presentation, neurobehavioural development, total cranial vault remodelling, craniosynostosis, sagittal craniosynostosis

## Abstract

Sagittal suture synostosis (SSS), caused by premature closure of the sagittal suture of the skull, is usually diagnosed and treated in the first few months of life; delayed diagnosis can be associated with abnormalities in brain development, including raised intracranial pressure (ICP) and neurocognitive development impairments. It can also affect an individual's self-perception and self-esteem. We present a unique case of late presentation and treatment of non-syndromic sagittal synostosis in a 10-year-old. Whilst the patient and his family’s main concerns were aesthetic, he also had neurobehavioural issues and needed glasses for vision. Total cranial vault remodelling was offered and successfully performed at the age of 10; this normalised his cephalic index, immediately improved vision, prevented the progression of neurobehavioural under-development and significantly improved self-esteem. This case highlights the difficulties of sagittal synostosis diagnosis, the potential consequences of delayed presentation and the success of treatment, even in an older age group.

## Introduction

Non-ossified zones, known as cranial sutures, separate the cranial bones at the beginning of life. The sagittal suture runs longitudinally from the frontal to the occipital bone, joining the two parietal bones. The sutures allow cranial deformation to promote safe passage through the birth canal, and remain patent during childhood to permit expansion upon brain growth. Intracranial volume increases rapidly within the first five years of life; by five years of age, the volume is 90% of the volume observed at 15 years. However, intracranial volume continues to increase until adulthood. The sagittal suture is the last suture to fuse, remaining patent until the fourth decade of life.

The premature fusion of one or more cranial sutures is a congenital condition known as craniosynostosis. Sagittal synostosis refers to the early fusion of the sagittal suture and accounts for 50-60% of all synostoses. It affects between 4,200 and 5,000 live births. Sagittal synostosis causes scaphocephaly and a reduced cephalic index [1]. Patients usually present in the first year of life after the unusual head shape is noticed by a parent or health visitor. Palpation of bony ridges at the affected suture can also signify sagittal synostosis during early neonatal check-ups.

Around 20% of cases of craniosynostosis are associated with a genetic syndrome and affect several sutures [2]. Mechanical deformation and foetal constraint have also been linked with the development of craniosynostosis. This has been supported by the increased prevalence of sagittal synostosis in twins, especially by the concordant development of sagittal synostosis in dizygotic twins [3]. Proposed (and widely accepted) foetal constraint factors include intrauterine compression and foetal positioning [4]. In addition, the mechanical factors of early foetal descent and cephalopelvic disproportion, resulting in foetal head constraint, may contribute to premature sagittal suture fusion. Currently, it is unclear whether the association is consequential or causative. However, research into cranial reshaping carried out during the Maya civilization suggests that increased external pressure may modify suture fusion [5].

Little is known about cases of craniosynostosis presenting above the age of six, and very few studies discuss long-term follow-up. However, the existing literature suggests that late presentation is a significant area for further exploration, as this case will demonstrate. In this case, the patient did not present until he was 10 years old, which had interesting implications for the manifestations and surgical correction of his sagittal synostosis. Whilst sagittal synostosis was initially considered a primarily aesthetic issue, this case also highlights the potential impact of a prolonged decrease in skull volume and delayed neurobehavioural development.

## Case presentation

A 10-year-old boy presented to the Oxford Craniofacial Unit unusually late, with an abnormal "egg-shaped" head, blurred vision, headaches and dyspraxia. He is the second child of unrelated parents. There was no history of undergoing any known in-utero mechanical stresses. Both the pregnancy and the vaginal delivery at term were uncomplicated. The patient has a background history of attention deficit hyperactivity disorder (ADHD), having been hyperactive since early childhood. He had been managed with methylphenidate and melatonin for 18 months prior to the surgery. Aside from this, he had no other medical conditions, was not on other medications and had no allergies. There is no family history of sagittal synostosis or other congenital/ craniofacial disorders.

The patient’s mother had been aware of the unusual head shape from birth, but he was not referred to the craniofacial clinic until she expressed significant concerns about bullying at school. 

Examination and treatment

**Figure 1:**
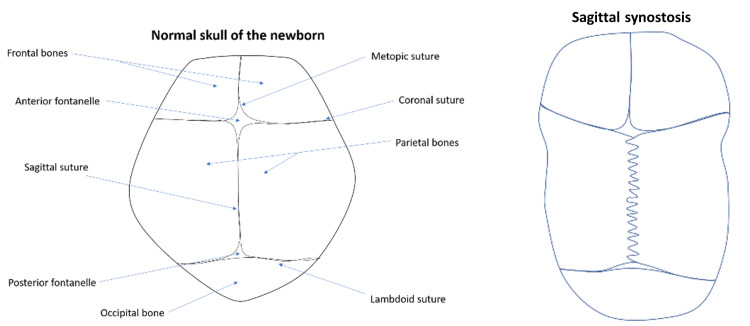
Comparison between normal skull and sagittal synostosis Clinical features of sagittal synostosis include sagittal ridging, restricted growth in biparieto-temporal areas and over-compensatory growth causing frontal bossing and occipital protrusion.

**Figure 2:**
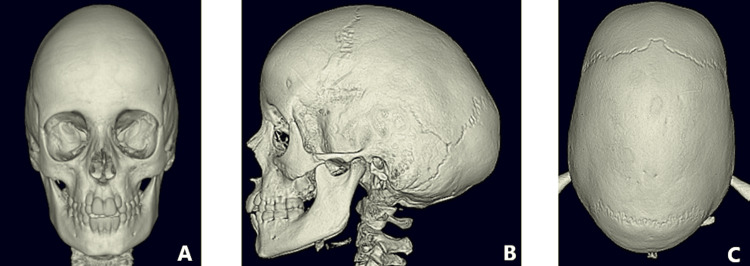
Pre-operation CT scans (A) Pre-operation anterior-posterior view CT; (B) Pre-operation lateral view CT; (C) Pre-operation vertex view CT

On examination prior to surgery, he had scaphocephaly (a "boat-shaped" skull), with a prominent forehead and occipital region (Figure 1). The routine genetic screening found no known mutations associated with craniosynostosis. A CT scan with three-dimensional reconstruction confirmed the non-syndromic sagittal synostosis (Figure 2). This is the gold standard for confirming a diagnosis of sagittal synostosis and has a diagnostic accuracy of 90-100%. Imaging showed marked copper beating and optic disc pallor (without papilloedema), however, the measured intracranial pressure (ICP) was normal at 7mmHg. He underwent total cranial vault remodelling using the lateral switch rotation technique at the age of 10. Cranial vault remodelling revises the fused suture and directly addresses the hypoplastic and compensatory cranial abnormalities, thereby offering predictable and stable results [6]. Post-surgical recovery was uncomplicated, and the patient was discharged from the hospital five days later. 

Outcome and follow-up

**Figure 3:**
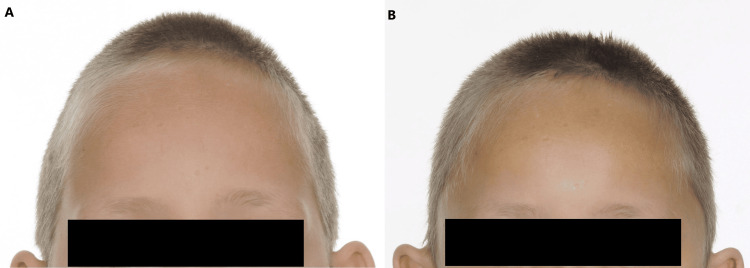
(A) Anterior preoperative image; (B) anterior postoperative image

**Figure 4:**
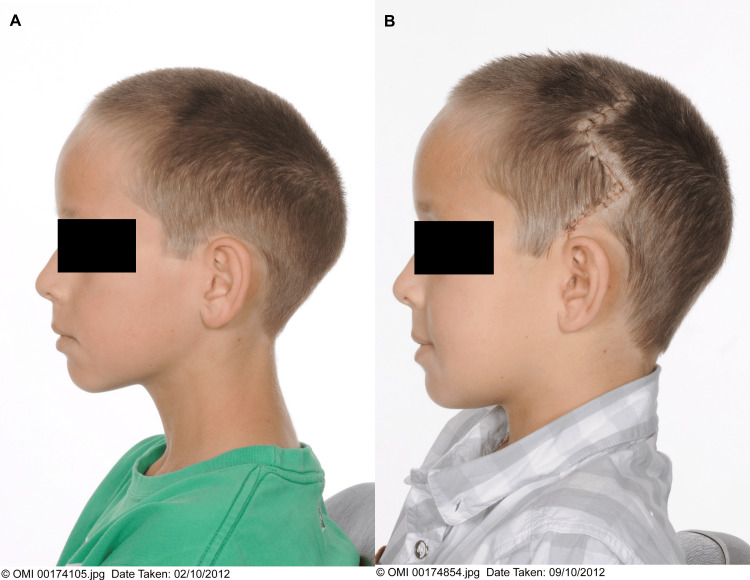
(A) Lateral preoperative image; (B) lateral postoperative image

**Figure 5:**
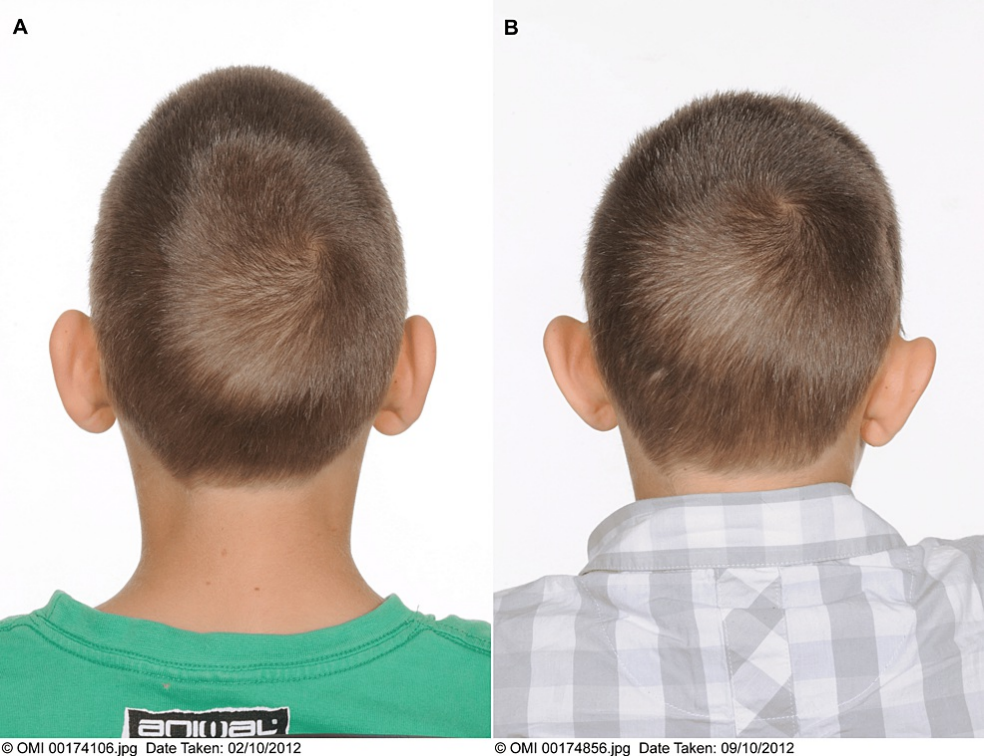
(A) Posterior preoperative image; (B) posterior postoperative image

The corrective surgery successfully fulfilled the main criteria: to prevent the progression of, and to correct the abnormality (Figures 3-5). He described waking up from the surgery with improved vision and the cessation of the "buzzing" noise in his head. A few months later, the patient required his glasses for only close work, having previously needed to wear them all day. The patient's dyspraxia also improved in the months post-surgery, and his dose of oral methylphenidate was initially halved from 36mg to 18mg. His melatonin prescription was stopped two years later. His school reported significant improvement in his behaviour and concentration. The psychosocial appearance benefits of the surgery are also notable. The patient described the surgery as "life-changing", and attributes his confidence and mental wellbeing to it. The craniofacial clinic followed him up for the remainder of his childhood. He was discharged from the craniofacial service after seven years, as he and his family were very pleased with the outcome of his surgery and the clinical team were satisfied that further complications were unlikely. 

## Discussion

Diagnostic difficulties

There are three important aspects of the patient's case. Firstly, it is a clear example of the difficulties of diagnosing sagittal synostosis in patients with scaphocephaly and later onset of other symptoms. It may indicate the benefit of further education about craniosynostosis for health visitors to promote early detection. This is especially important in cases, such as this patient's, where there is an unknown genetic confounder or idiopathic cause. The only known risk factor in the patient's case is his sex; sagittal synostosis is four times more common in males. It has been suggested that this is due to the faster growth (and larger size) of the male foetal head during the third trimester, which might increase the risk of deformation secondary to constraint [7]. 

The role of genetics in non-syndromic sagittal synostosis is a recent finding, in contrast to the well-accepted association between genetic mutations and coronal or multiple suture synostosis. A mutation in SMAD6, which is required for intracellular signal transduction by bone morphogenetic protein (BMP), has been discovered in 3% of patients with sagittal synostosis, and 6% of individuals with scaphocephaly but no craniosynotosis [8]. However, like the patient, many cases of sagittal synostosis cannot be linked to known in-utero mechanical stress and have no known SMAD6 mutation. Current evidence suggests that the aetiology may be multifactorial in many cases, arising from polygenic factors interacting with epigenetic and environmental factors during perinatal life [9]. Therefore, cases such as the patient are particularly notable in emphasising the importance of considering sagittal synostosis in cases of scaphocephaly where there is no clear aetiology. 

Delayed presentation

Secondly, whilst the causative association between sagittal synostosis and scaphocephaly has been highlighted in other cases, it is very rare for a patient to present this late. There is an association between sagittal synostosis and raised ICP, which has only recently been accepted. This is fitting with the Monro-Kellie hypothesis: the fused cranium restricts brain expansion, and therefore ICP increases. The patient subjectively reported headaches, tinnitus and blurred vision for many years prior to his surgery, which all resolved immediately post-operatively. These symptoms are all associated with raised ICP, which was also suggested by the marked copperbeaten appearance of the skull on CT and fundoscopy findings of optic disc paling. However, the ICP was found to be 7mmHg in a pre-surgical measurement. Therefore, patients with scaphocephaly can have both subjective and objective features which are suggestive of raised ICP, despite normal ICP measurements; whilst this is reassuring, it does not mean that surgery is unnecessary or not beneficial, as highlighted by this case. 

The case highlights that whilst the literature may be primarily focused on the physiological consequences of sagittal synostosis, sagittal synostosis may also affect neurobehavioral development, as indicated by the patient's dyspraxia, poor motor control and attention deficit. This case supports studies that have suggested that while older children with sagittal synostosis may have an average global IQ, it is frequently accompanied by neurocognitive or neurobehavioral impairments such as learning difficulties, language impairment, coordination or attention deficit. This suggests that the global delays seen in younger children may develop into specific forms of impairment in later childhood. Moreover, there is little assessment of specific function areas, such as attention or inhibitory control, in younger children, so there may also be unidentified functional deficits during infancy. The unreliability and imprecision of infant cognitive tests mean it is essential to follow up with children throughout their school life. 

It is unknown whether the delay in neurobehavioural development is a result of raised ICP, primary brain deformation or secondary brain deformation due to brain growth in an abnormally shaped cranium [10]. Three-dimensional imaging has indicated that some patients may have morphologic abnormalities in brain structure, including small frontal lobes, displacement of the lateral ventricles and a compressed corpus callosum [11]. This may explain some of the neurocognitive abnormalities, as these areas have been associated with the development of learning, language, coordination and attention disorders. Frontal lobe abnormalities in particular have been associated with impairments in executive functions seen in older children with sagittal synostosis [12]. The association between the patient’s ADHD and sagittal synostosis is unknown; interestingly, the patient’s hyperactivity improved dramatically in the months following surgery, and the difference in his behaviour and concentration was noted both at school and at home. This allowed his methylphenidate dose to be halved. However, two years after the surgery his methylphenidate dose was increased again, although his melatonin was stopped. Notably, however, the patient’s dyspraxia (which had been diagnosed at the age of five) completely resolved in the months following his surgery. Currently, there is little specific research into the association between non-syndromic sagittal synostosis and dyspraxia, but this is a potential area for further exploration. 

The impact of scaphocephaly on sagittal synostosis patients should not be trivialised. Patients who present later in childhood have often experienced bullying at school, which can have detrimental effects on their self-esteem. Often, this does not start until the latter years of primary education, supporting the theory that children aren't fully aware of differences in appearance until the age of seven [13]. Therefore, this could arguably have been avoided by earlier intervention. 

Success of surgery

Finally, the case highlights the value of cranial vault remodelling, despite the increased risk and potentially lower efficacy associated with the patient's older age. Typically, children undergo surgery between six months and two years, which utilises this period of rapid brain and cranial growth. This optimises the chance of re-ossification and bone remodelling, and so reduces the need for bone grafting. Surgical correction becomes difficult in older children, as the cranium is thickened and harder to remodel. However, the patient's surgery had a significant psychosocial appearance benefit and normalised the defects seen on his cranial imaging; his cephalic index was corrected from 66% to 78%. 

Furthermore, his neurocognitive impairment improved, strengthening the case for research into the impact of surgery on neurobehavioural development. Some studies have suggested that there is a positive correlation between early surgery and cognitive performance [14]. A possible hypothesis is that delaying surgery prolongs the exposure to complications of a reduced skull volume and compromises brain development; this has been supported by the post-surgical improvements in mental function in patients with a pre-operatively increased ICP. However, larger sample sizes are required to confirm this association, as other studies investigating age at surgery and mental function have shown positive correlations but have not had significant findings [15]. 

The immediate post-operative improvement in the patient’s vision is an area for potential future research. Previous studies have shown an association between craniosynostosis and visual impairment, such as hypermetropia, astigmatism and strabismus, which may lead to amblyopia if uncorrected [16]. However, there are few studies which relate specifically to non-syndromic sagittal synostosis. There is also debate about whether early surgical intervention can improve vision. Visual development has a critical phase from infancy until its completion at around eight years of age and can be disrupted by significant refractive error or lack of alignment or coordination. Some research has suggested that if these conditions aren’t detected and treated during this critical period of visual cortex plasticity, there will be a permanent effect on visual function. This is supported by a study which concluded that most children who underwent surgery later than six months had considerable astigmatism, and therefore early surgery is key to minimising the risk of developing visual problems [17]. However, subsequent research has disagreed with this, suggesting that operating on children between 12 to 18 months of age resulted in a lower percentage of refractive error/squint than operating before 12 months [18]. This case may provide additional support for late surgical intervention to improve vision, although further thorough research is required into this area. 

The case also identifies other areas for possible future investigation, for instance, there is very little research into the association between non-syndromic sagittal synostosis and dyspraxia. 

## Conclusions

This case highlights several key learning points relating to the delayed presentation and treatment of sagittal synostosis. It emphasises that sagittal synostosis can present with no clear risk factors and an absence of raised ICP, and therefore may draw attention to an important learning point for health visitors and primary care practitioners. Furthermore, the case suggests that late presentation of sagittal synostosis may result in delayed neurobehavioural development and impaired vision, and give rise to psychosocial difficulties. This highlights the necessity of early diagnosis. However, a crucial learning point is that corrective surgery in late childhood can still be very successful in normalising the cephalic index, improving vision, preventing the progression of neurobehavioural under-development and improving self-esteem. 
